# Atmospheric electroaerodynamic thrusters with grid collectors

**DOI:** 10.1038/s41598-025-25308-6

**Published:** 2025-11-24

**Authors:** Raffaello Terenzi, Stefano Trovato, Davide Usuelli, Marco Belan

**Affiliations:** Department of Aerospace Science and Technology, Politecnico di Milano, Milan, 20156 Italy

**Keywords:** Atmospheric ion thruster, Corona discharge, EAD propulsion, Grid collectors, Aerospace engineering, Applied physics, Plasma physics

## Abstract

The present study investigates the effects of adopting grid structures as collector electrodes on the performance of electroaerodynamic (EAD) thrusters. A systematic experimental campaign was carried out to determine how key geometric parameters of the grids—including spatial density and wire diameter—impact thruster performance. Dimensionless coefficients were introduced in order to enable meaningful comparisons with respect to existing literature. The results reveal the existence of an optimal grid configuration, leading to a significant enhancement in performance compared to the state of the art for a single stage unit, such as in thrust density, achieving a maximum of $7.03 \ \mathrm {N/m^2}$ at $20 \ \textrm{kV}$ of applied voltage, and thrust-to-weight ratio, reaching a maximum value of 9.51, all achieved without compromising the propulsive efficiency typical of traditional EAD thrusters.

## Introduction

The growing interest of the aviation industry in electric propulsion systems, dictated by a more sustainable transport policy, has directed interest towards alternative technologies capable of meeting the increasingly stringent regulations. Among the various solutions, alongside the well-established application of electric motor-driven propellers^1,2^, electroaerodynamic propulsion (EAD) has emerged as a promising candidate. Although plasma propulsion is widely utilized in the space sector^3–5^, plasma thrusters—characterized by their simple geometries, absence of moving mechanical components and minimal noise emissions—have only recently begun to be explored for atmospheric flight applications, in the form of EAD propulsion, yet showing significant promise. Over the past decade, the feasibility of EAD propulsion for sustained flight has been assessed^6^ and demonstrated, with notable milestones including the development of ion-powered aircraft^7,8^ and ionocraft^9,10^. In its simplest configuration, an EAD thruster consists of two electrodes powered by a high voltage source. The first electrode, commonly referred to as the emitter, typically consists of a wire with a diameter smaller than $200 \upmu \textrm{m}$, though alternative geometries including pins and blades^11–16^ are also reported in the literature. The second electrode, known as the collector, is characterized by a larger cross-sectional area and in the past has been implemented in different shapes—from cylinders to streamlined bodies including droplets and airfoils^17–19^. These configurations have been the focus of optimization studies aimed at enhancing overall performance^19–21^. When a high voltage is applied between the electrodes, an intense electric field is generated to the point of promoting the ionization of the air in the vicinity of the emitter. The resulting ionized air, driven by the electric field, travels toward the collector through the so-called drifting region: during the motion, ions transfer momentum to neutral air molecules via collisions, thereby generating a net thrust. The performances of EAD thrusters are typically evaluated considering, in addition to the mere thrust generated, the thrust-to-power ratio, an index of system efficiency, and the thrust density, which refers to the compactness of the thruster. Dimensionless coefficients have been introduced^21,22^ in order to provide meaningful comparison between different thruster configurations. Over the years, extensive research has been carried out to improve several aspects that contribute to the generation of thrust in order to enhance performance indicators. Among these, different plasma generation techniques have been explored. While the corona discharge regime, powered by a direct current (DC) source, remains the most employed method, alternative approaches such as dielectric barrier discharge (DBD)^18,23^ and nanosecond pulsed power supplies^24,25^—both relying on alternating currents (AC)—have also been investigated. The simplicity of EAD thrusters manufacture due to the minimal component requirements facilitates modifications to the geometric parameters that define the thruster. This adaptability has led to studies exploring diverse configurations, including the effect of the emitters arrangement^26,27^, as well as the possibility of stacking the individual propulsion units either vertically or in series^19,20,28,29^, which demonstrated significant performance improvement compared to single unit configurations. Recently, wind tunnel tests have been carried out to assess EAD thrusters performance in a controlled airflow environment^30–33^, while other experimental studies have investigated the influence of pressure, temperature and humidity on their performance^34–36^. Nevertheless, despite the progress made and the intrinsic advantages of the EAD propulsion principle, current implementations still present clear limitations in terms of thrust-to-power ratio and thrust density, when compared with contemporary propulsion technologies. The present work addresses these limitations by investigating the use of grid structures as collector electrodes to enhance overall thruster performance while significantly reducing system weight and volume. The results from an extensive experimental campaign are presented, where different grids have been tested to evaluate performance trends based on geometric parameters including wire thickness and spacing. This work is organized as follows: in Section "Design of experiment" the design of experiment together with the derivation of the performance coefficients are described. Section "Experimental setup" presents the experimental setup and the measurement techniques. In Section "Results" the results obtained are shown while Section "Conclusions" presents the conclusions.

## Design of experiment

### Grids parametrization

The aim of the present study is to identify the propulsive performance trends of EAD thrusters employing grids as collectors. The motivation behind the choice of these electrodes lies in the ability of a grid to approximate the electrostatic behavior of an equipotential plate. This configuration could generate a pronounced asymmetry in the electric field, thereby enhancing performance in the corona regime. This effect becomes more significant as the collector density increases (approaching the limiting case of a solid plate, characterized by the shielding phenomenon^27,37^); however, the corresponding increase in aerodynamic resistance, due to a higher density of the collector plane, is expected to degrade the propulsive performance. As a result, the presence of an optimum in performance is expected, arising from the opposing influences of the two principal physical phenomena governing EAD thruster operation: namely the electric and aerodynamic effects.

**Fig. 1:**
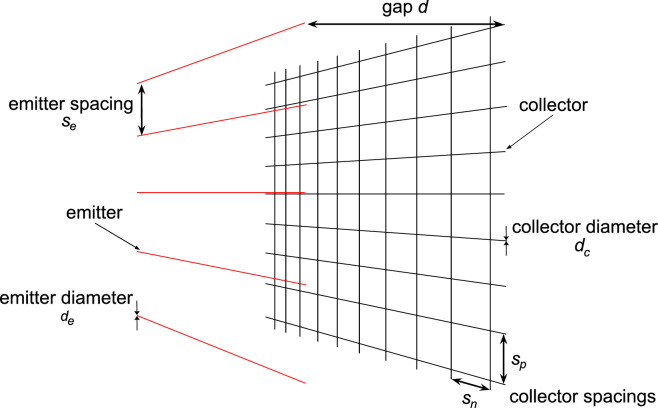
Schematic sketch of an EAD thruster with grid collectors and main geometric variables (not to scale).

To characterize the grid configurations tested in this work, a geometric parameterization is introduced for both the emitter and the collector electrodes. Specifically, in a thruster designed to simulate periodic conditions, the most relevant geometric parameters for both electrodes are the wire diameter and the spacing between adjacent wires (Fig. 1).

These parameters can be combined into a single variable—one for each electrical polarity—referred to as the grid density, defined for the emitter and collector respectively as:

1 $$\begin{aligned} \rho _e= & \frac{d_e}{s_e} \end{aligned}$$

2 $$\begin{aligned} \rho _c= & d_c \left( \frac{1}{s_p} + \frac{1}{s_n} \right) \end{aligned}$$

where $d_e$ and $s_e$ denote the emitters diameter and spacing, respectively, while $d_c$, $s_p$ and $s_n$ correspond to the collector wire diameter and spacings in the directions parallel and normal with respect to the emitters orientation. The introduction of collector wires arranged orthogonally to the classical configuration enables a significant increase in grid density, thereby expanding the range of tested configurations. The quantities $\rho _e$ and $\rho _c$ represent the fraction of the thruster’s total frontal area that is occupied by the electrodes. It should be noted that the definition of $\rho _c$ does not account for the intersections between collector wires arranged in parallel and normal directions. As a result, the calculated density in these configurations is slightly overestimated. However, given the small diameter of the wires employed in this study and the densities limited to less than 0.16, the contribution of these intersections to the overall frontal area introduces a maximum error below $4\%$, which is negligible when compared with the variations in $\rho _c$ discussed in this work.

This study primarily focuses on geometries confined within the electrode planes. To assess their influence on thruster performances, variations in both diameters and spacings are systematically explored, individually and in combination. Specifically, four distinct configuration groups are defined based on the emitter and collector diameters, as summarized in Table 1. A comparison between these groups allows for evaluating performance trends as functions of emitter and collector diameters, $d_e$ and $d_c$, respectively. Within each group, the influence of emitter spacing $s_e$ and collector spacings $s_p$ and $s_n$ on thrust density *T*/*A* and thrust-to-power ratio *T*/*P* is analyzed. Only uniform spacings are considered in this study, with the available values corresponding to those reported in Table 2. Not all the presented combinations were tested: a limited number of dual configurations—where $s_p$ ad $s_n$ could be interchanged—were analyzed in order to assess the effect of wire orientation for a given $\rho _c$, which alone does not allow differentiation between the two layouts. Certain configurations have been deliberately excluded, specifically those with $s_e$ being smaller than $s_p$ and $s_n$ to ensure proper electric field asymmetry across the electrodes, thus mitigating the detrimental effect of reverse corona. In addition, large $s_e$ values were omitted from some configurations, as preliminary tests indicated they were unlikely to deliver promising performance. In the context of this work, an infinite collector spacing (i.e., $s_n \rightarrow \infty$) indicates the absence of wires in that particular orientation. The distance between emitter and collector planes *d* is held constant, as well as the applied voltage, as their impact on thrust and electrical characteristics has been thoroughly examined in previous studies^38,39^.

**Table 1:** Configuration groups and their respective electrodes diameters.

Configuration	$d_e \ [\textrm{mm}]$	$d_c \ [\textrm{mm}]$
I	0.03	0.1
II	0.1	0.1
III	0.03	0.03
IV	0.03	0.2

**Table 2:** Available electrodes spacings.

$s_e \ [\textrm{mm}]$	7.5	10.0	12.5	15.0	17.5	22.5	32.5	47.5	77.5
$s_p, \ s_n \ [\textrm{mm}]$	1.25	2.5	7.5	12.5	17.5	22.5	$\infty$		

### Dimensionless coefficients

The performance of the gridded EAD thruster is evaluated in terms of thrust density $T/A$, an index of compactness, and thrust-to-power ratio $T/P$, which accounts for the thruster efficiency. To enable a meaningful comparison between both the tested configurations and those reported in the literature^21,22^, their corresponding dimensionless parameters are introduced as $C_{TA}$ and $C_{TP}$, respectively, allowing performance to be assessed in terms of compactness and efficiency, regardless of its location along the well-established applied-voltage and gap characteristic curves, and of the physical dimensions arising from a specific experiment. The complete derivation can be found in the work^22^, here the dimensionless coefficients are directly reported for brevity. Thrust density coefficient $C_{TA}$, is defined as follows:

3 $$\begin{aligned} C_{TA} = \frac{T}{A} \frac{d^2}{\epsilon _0 {V_c}^2} \end{aligned}$$

where *T* denotes the generated thrust, *d* is the gap, $\epsilon _0$ is the vacuum permittivity equal to $8.85 \times 10^{-12}~\mathrm {C/Nm^2}$, *A* is the reference area and $V_c$ is the voltage across the electrodes.

Thrust-to-power ratio coefficient $C_{TP}$ is defined as:

4 $$\begin{aligned} C_{TP} = \frac{T}{P} \frac{\mu _q V_c}{d} \end{aligned}$$

where $\mu _q$ is the ion mobility expressed as $\mu _q=2.5 \times 10^{-4}~\mathrm {m^2/sV}$ and $P = V_c \ I_c$ corresponds to the electrical power consumed across the electrodes.

Additionally, a dimensionless form is introduced for the corona current $I_c$—as the current drawn by the thruster serves as an indicator of proper operation—resulting in the $C_C$ current coefficient with the following definition:

5 $$\begin{aligned} C_C = \frac{I_c}{I_{MG}} \end{aligned}$$

where $I_{MG}$ corresponds to the Mott-Gurney current^40^ expressed as $I_{MG}=\frac{9}{8} \epsilon _0 \mu _q A V^2/d^3$ which—although its validity does not hold in the case of the corona discharge regime—is commonly employed as a reference value for scaling the current density^22,39^. The current coefficient $C_C$ could be used to determine the nominal operation of the thruster. High values of $C_C$ are generally associated with the phenomenon of reverse corona, an undesirable effect leading to a significant increase in power consumption due to ionization occurring also in proximity of the collector, which therefore does not contribute to thrust generation. In this test campaign, gap *d*, applied voltage (equal to $20 \ \textrm{kV}$) and frontal area *A* are kept constant.

## Experimental setup

**Fig. 2:**
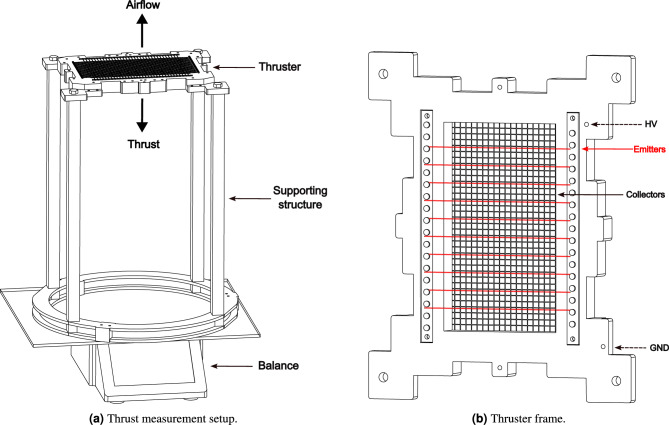
Illustration of the experimental setup.

An experimental setup, similar to those used in previous studies^16,21,26^, was designed and built using Acrylic Styrene Acrylonitrile (ASA) through rapid prototyping to allow electrical and thrust measurements. The complete setup, illustrated in Fig. 2a, consists of two main components: the thruster frame and the supporting structure. A detailed view of the EAD thruster is shown in Fig. 2b. The frame, designed to house the electrodes within a $100\times 175 \ \textrm{mm}$ frontal area, is a single rigid component capable of preserving a fixed gap *d* of $20 \ \textrm{mm}$ between the two electrodes planes. Wires are wrapped around pegs as a serpentine creating a grid-like geometry. On the collector side, two sets of parallel arrays of pegs allows to arrange wires both parallel and normal to the emitters direction. Two arrangements are available: pegs with a diameter of $2.5 \ \textrm{mm}$ evenly spaced at $5 \ \textrm{mm}$ or pegs with diameter of $1.25 \ \textrm{mm}$ evenly spaced at $2.5 \ \textrm{mm}$ to get higher values of $\rho _c$. In this study, three different wire diameters are employed (0.03, 0.1 and $0.2 \ \textrm{mm}$), increasing the number of possible tested configurations, as stated in Section "Design of experiment". The frame is designed to mitigate edge effects, which are typically caused by intensified electric fields at the boundaries of the spatial domain. The supporting structure transfers the thrust load to the balance while keeping the frame at a distance of $400 \ \textrm{mm}$ from the balance plate, thus ensuring the absence of electrostatic interference which is further mitigated by grounding the plate itself.

### Thrust measurements

The net propulsive force generated by the thruster is measured using a high-precision balance with an accuracy of $0.1 \ \textrm{mN}$ and a full-scale capacity of $40 \ \textrm{N}$. The force is determined by calculating the difference between the thruster being switched on and off, respectively. The ionic wind flows in the upward direction and the electrical connections are designed and arranged to be minimally intrusive in terms of length and cross-section, ensuring that the measurement remains unaffected by fluid dynamic, mechanical and electrical interference.

### Electrical measurements

**Fig. 3:**
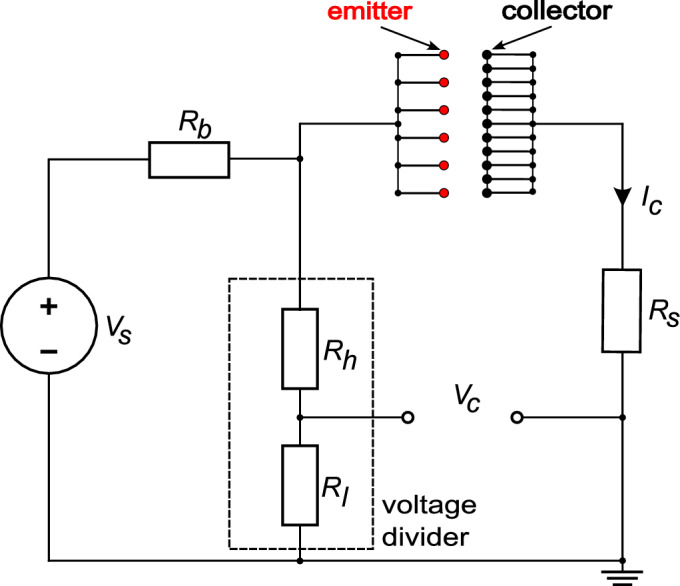
Schematic of the electric circuit.

In this test campaign the thruster always operates under positive corona discharge regime, with the emitters connected to the high voltage source while the collectors are electrically grounded. During testing, voltage and current measurements are measured across the electrodes to evaluate propulsive efficiency. The electrical scheme of the experimental setup is shown in Fig. 3. A high-voltage power supply $V_s$, capable of delivering up to $20 \pm 0.03 \ \textrm{kV}$, provides the necessary potential difference between the emitters and collectors. A ballast resistor $R_b=1.0 \pm 0.001 \ \mathrm {M\Omega }$ is included in the circuit for safety purposes. Voltage measurements are obtained using a voltage divider (ratio 1 : 1000) with an impedance of $R_{tot}=R_h+R_l=47 \pm 0.001 \ \mathrm {M\Omega }$, placed in parallel with the thruster. Measurements of the corona current $I_c$ are obtained from the voltage drop across a shunt resistor $R_s=88.5 \pm 0.01 \ \mathrm {\Omega }$ positioned in series with the thruster.

### Data acquisition

Thrust values are directly read from the balance, while electrical measurements ($V_c$ and $I_c$) are acquired using an oscilloscope with an observation time of $T_{obs}=2$ seconds and a sampling frequency of $f_s=50 \ \mathrm {ks/s}$. For each test, twenty acquisitions are performed and results are averaged. The statistical analysis is based on the time histories of each individual test, while the final uncertainties in voltage, current and thrust are obtained by combining the results of the multiple acquisitions using the Root Sum of Squares (RSS) method.

## Results

### Effect of emitter and collector spacings

**Fig. 4:**
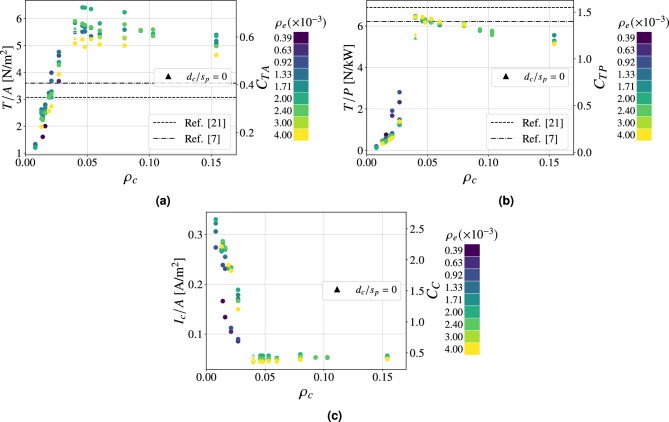
Performance parameters as a function of $\rho _c$ at $20 \ \textrm{kV}$ of applied voltage for Configuration I. $95\%$ confidence intervals (mean values): **(a)** $\pm 0.10 \ \mathrm {N/m^2}$, **(b)** $\pm 0.16 \ \mathrm {N/kW}$, **(c)** $\pm 0.004 \ \mathrm {A/m^2}$. $\bullet : \textrm{finite} \ s_p$, $\blacktriangle : s_p \rightarrow \infty$.

**Fig. 5:**
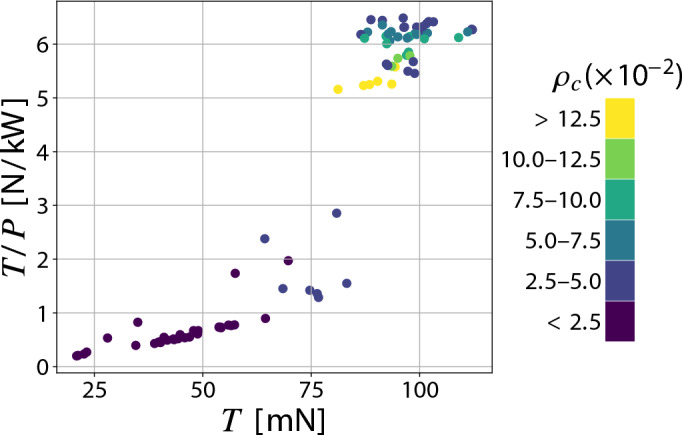
Thrust-to-power ratio against thrust as a function of collector density $\rho _c$. $95\%$ confidence intervals (mean values): *T*/*P*: $\pm 0.16 \ \mathrm {N/kW}$, *T*: $\pm 4 \ \textrm{mN}$.

The foundation of this study is the analysis of a configuration featuring emitters with a diameter of $0.03 \ \textrm{mm}$ and collectors with a diameter of $0.1 \ \textrm{mm}$, referred to as Configuration I (Table 1). This configuration was selected based on a trade-off between performance and mechanical robustness. A smaller emitter diameter reduces the ignition voltage, thereby enhancing performance at a fixed applied voltage and improving the signal-to-noise ratio. Conversely, a larger collector diameter contributes to improved mechanical stability and durability. Figure 4 shows the performance parameters as a function of the spacings $s_e$, $s_p$ and $s_n$ expressed as grid densities $\rho _e$ and $\rho _c$ for the mentioned configuration. For comparison, reference configurations representative of the established and current state of the art are provided^7,21^. In the latter case, they correspond to streamlined airfoil-like collectors with different chord lengths $c=25, \ 20$ $\textrm{mm}$ and thickness-to-chord ratio $t/c=0.24$. For each value of collector grid density, multiple tests were conducted varying $s_e$, as previous studies have shown that, for a given collector configuration in a finite-sized thruster, an optimum in performance exists due to both the number and the arrangement of the emitters^21,26,27^. This behavior arises from the interplay of two effects as emitter density increases: on one hand, a higher anode density enhances ion generation; on the other hand, it promotes shielding, which delays the emitters ignition at a given supply voltage^26,27,37^. As shown in Fig. 4a, the thrust density increases with collector density, reaching a maximum dimensionless value of 0.73 (equal to $6.4 \ \mathrm {N/m^2}$ for an applied voltage of $20\ \textrm{kV}$) for $\rho _c\approx 0.04$ and $\rho _e\approx 0.003$, which corresponds to an absolute emitters spacing of 15 $\textrm{mm}$, before decreasing as collector density further grows. The reduction in performance at higher densities is attributed to the elevated number of wires: although this brings the configuration closer to the case of a solid plate, it also leads to greater aerodynamic resistance, thereby reducing thrust. It is noteworthy that, starting from the plateau region (i.e., for $\rho _c \ \ge 0.04$), configurations with equivalent grid densities exhibit comparable thrust density values, regardless of wire orientation (Fig. 4a). In the case presented, a collector density of $\rho _c=0.04$ can be achieved either by using only wires parallel to the emitters (i.e., $s_p=2.5 \ \textrm{mm}$ and $s_n \rightarrow \infty$) or only wires perpendicular to them (i.e., $s_n=2.5 \ \textrm{mm}$ and $s_p \rightarrow \infty$)—two contrasting configurations that serve as representative cases. The maximum difference observed in thrust density among all tested configurations with $\rho _c=0.04$ and identical emitters arrangement is approximately $4\%$. This finding supports the hypothesis that collector wires mutually shield each other, thereby reducing the local electric field intensity and making the wire orientation effectively irrelevant. Within the operational range, this justifies the use of the unified collector parameter $\rho _c$, even though it does not distinguish between different wire orientations. It is important to highlight that the maximum thrust density value achieved with these configurations is significantly higher than that reported in the reference study^21^. In that case, the optimal dimensionless value corresponds to 0.34, implying an improvement of approximately $114 \%$. The performance improvement is also evident when expressing the most efficient configuration in dimensional terms: scaled to an applied voltage of $20 \ \textrm{kV}$, it yields a thrust density of $6.4 \ \mathrm {N/m^2}$, corresponding to an increase of approximately $78\%$ compared to the MIT aircraft^7^. Figure 4b shows the effect of grid density on the thrust-to-power ratio. Similar to the thrust density behavior, the dimensionless performance parameter increases with collector density up to a maximum of 1.47 at $\rho _c = 0.04$ (yielding a thrust-to-power ratio of $6.52 \ \mathrm {N/kW}$ for $20 \ \textrm{kV}$ of applied voltage), then declines as the density increases further. The underlying cause of this behavior is the onset of reverse corona for lower collector density configurations. This phenomenon, triggered by insufficient asymmetry in the electric field between the emitters region and collectors, results in a stronger electric field in proximity to the collector, which initiates negative corona discharge. Igniting at a lower voltage than positive corona in symmetric configurations, negative corona generates ionic wind in the opposite direction to the desired thrust. The outcome results in a significant increase in current, summing the positive and negative corona contributions, and a corresponding decrease in thrust, both of which drastically reduce the thrust-to-power ratio. This interpretation is confirmed in Fig. 4c, which shows that configurations with lower collector density values exhibit substantially higher currents compared to cases where $\rho _c > 0.04$, with differences ranging over a full order of magnitude among the tested configurations. Moreover, the plot reveals that at higher wire concentrations the current remains approximately constant, hinting that the performance loss at high collector densities is primarily due to reduced thrust caused by increased aerodynamic resistance. This specific regime is identified as the plateau region. The decrease in propulsive force for greater collector densities—distinctively observable from $\rho _c > 0.125$—is ultimately confirmed in Fig. 5, which presents in dimensional form the impact of collector grid densification $\rho _c$ on both the net thrust and the thrust-to-power ratio. Furthermore, it can be noted how a sharp increase in thrust-to-power ratio—at collector densities around 0.04—accompanies only a gradual rise in thrust with increasing $\rho _c$, revealing two distinct operational regions in terms of efficiency, thus enabling a more precise identification of the optimal thruster envelope (i.e., the upper-right corner).

**Fig. 6:**
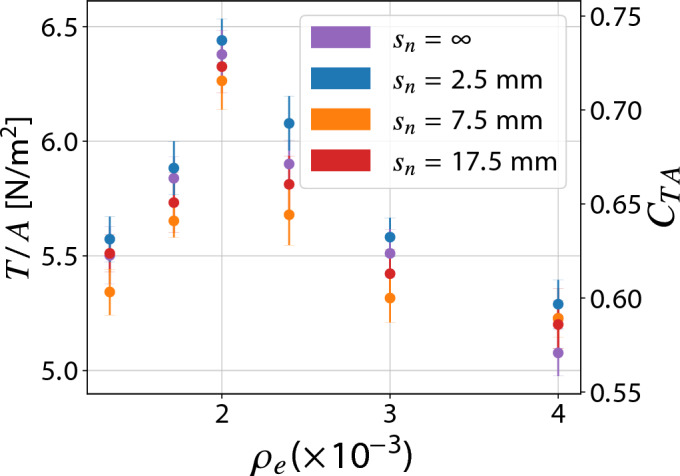
Thrust density as a function of the emitter’s mesh density $\rho _e$ and normal spacing $s_n$ (Configuration I, $s_p=2.5 \ \textrm{mm}$).

Figure 6 illustrates, to a greater extent, the influence of the emitter density $\rho _e$—obtained by varying $s_e$—on the thrust density. For simplicity, only the results obtained from the configurations with $s_p = 2.5 \ \textrm{mm}$ and varying $s_n$ are shown, which are representative of the phenomenon in question as they lie within the plateau. The same reasoning applies to other spacings. It is possible to notice how, for the different spacings in the normal direction $s_n$, an optimum as a function of the density of the emitters is encountered, in this case for $\rho _e$ equal to 0.002, which corresponds to the case with $s_e=15 \ \textrm{mm}$. As already mentioned above, the reason can be attributed to the compromise between the increase of ion-generating regions for the given frontal area and their mutual shielding effect—which is detrimental in the case of the emitters. It is therefore clear that the thrust density depends on the densities of both the emitting and collecting electrodes.

### Effect of electrodes diameters

**Fig. 7:**
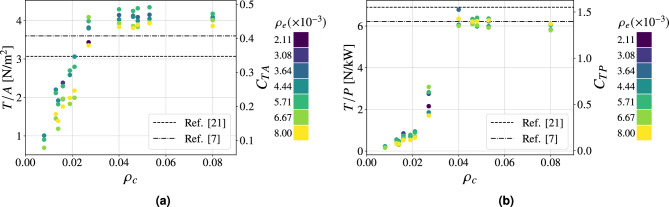
Performance parameters as a function of $\rho _c$ at $20 \ \textrm{kV}$ of applied voltage for Configuration II. $95\%$ confidence intervals (mean values): **(a)** $\pm 0.09 \ \mathrm {N/m^2}$, **(b)** $\pm 0.12 \ \mathrm {N/kW}$.

**Fig. 8:**
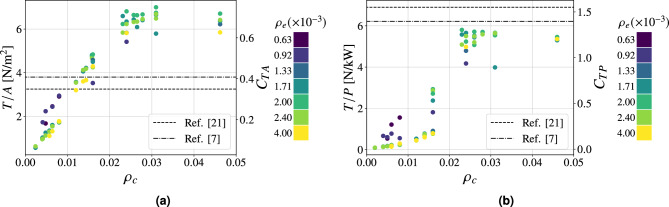
Performance parameters as a function of $\rho _c$ at $20 \ \textrm{kV}$ of applied voltage for Configuration III. $95\%$ confidence intervals (mean values): **(a)** $\pm 0.12 \ \mathrm {N/m^2}$, **(b)** $\pm 0.16 \ \mathrm {N/kW}$.

**Fig. 9:**
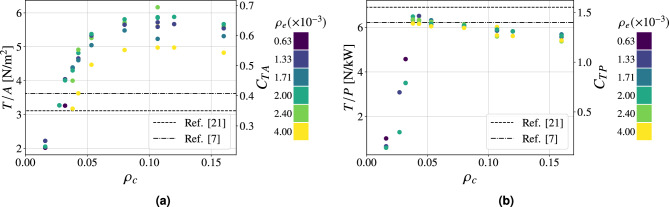
Performance parameters as a function of $\rho _c$ at $20 \ \textrm{kV}$ of applied voltage for Configuration IV. $95\%$ confidence intervals (mean values): **(a)** $\pm 0.09 \ \mathrm {N/m^2}$, **(b)** $\pm 0.14 \ \mathrm {N/kW}$.

To further investigate the influence of the emitter on the performance of the thruster, additional tests were performed using emitters with the same diameter as the collectors (i.e., $d_e = 0.1 \ \textrm{mm}$) referred to as Configuration II (Table 1). The results are reported in Fig. 7. The overall behavior of the thrust density for the larger emitter configurations (Fig. 7a) is similar to that of the $0.03 \ \textrm{mm}$ emitters, but with a lower maximum value (from $6.4 \ \mathrm {N/m^2}$ down to $4.3 \ \mathrm {N/m^2}$ in the present case). Indeed, the use of larger emitters (while keeping the same collectors as in Configuration I) results in lower thrust generation, due to a delay in the onset of positive corona to higher voltage values. Interestingly, the thrust-to-power ratio (Fig. 7b) remains relatively unaffected by the change in emitter diameter and both configurations exhibit similar behavior. This can be attributed to the fact that as larger emitters produce less thrust due to the diminished asymmetry of the configuration, they also consume less current, resulting in comparable thrust-to-power ratios. A similar analysis was performed on the collector, varying the diameter of its wires to 0.03 and $0.02 \ \textrm{mm}$, respectively identified as Configuration III and IV. The corresponding performance figures are shown in Fig. 8 and Fig. 9.

**Fig. 10:**
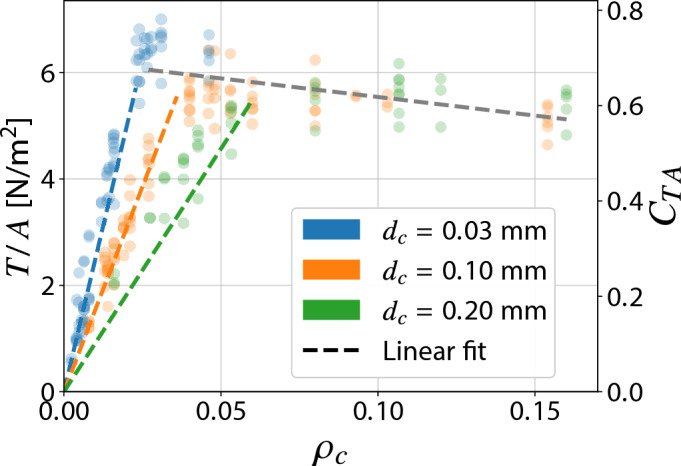
Thrust density as a function of the collector’s mesh density $\rho _c$ for Configurations I, III and IV.

The peak value for Configuration III is found for approximately $\rho _c = 0.031$ (with $\rho _e$ equal to 0.002), lower than the configuration with collectors with a diameter of $0.1 \ \textrm{mm}$. In contrast, for collectors with a diameter equal to $0.2 \ \textrm{mm}$, namely Configuration IV, the plateau is reached for $\rho _c$ equal to 0.08. The reason lies in the fact that reaching the plateau indicates that the electrical behavior of the grid is commensurate with that of a flat plate, which would be the optimal configuration from an electrostatic point of view. Indeed, it would ensure maximum asymmetry of the electric field between the emitters and the collectors, lowering the magnitude of the electric field near the collector to the lowest possible value and thus inhibiting any possible reverse corona. A possible qualitative index to describe this effect is the total number of collector wires for a given thruster cross-sectional area, which corresponds to the number of points where the voltage *V* is imposed by the actual presence of a grounded conductor, thus altering the local electric field. The spatial disposition of the wires is expected to have a second-order effect compared to the grid density itself, while still being relevant when comparing collector wires of different diameters. Under this hypothesis, using thinner wires for the same number of conductors results in a lower $\rho _c$, providing a heuristic explanation for the observed plateau at lower densities. Furthermore, it can be observed in Fig. 10 how, as the diameter of the collectors increases, the maximum achievable thrust density value decreases slightly (symbolized by the black dashed line). This effect, scarcely observable, is certainly a consequence of the higher grid density at the beginning of the plateau, which entails a higher aerodynamic drag. The inclination of the regression line at the plateau is representative of the drag coefficient of the collector wires, while the grid density itself is representative of the wetted surface. Their combined effect yields the aerodynamic drag, which is proportional to both the drag coefficient (approximately constant for all the wires, hence lying on the same regression line) and the wetted surface, being entirely responsible for the reduced thrust at higher grid densities. The data points for the thrust density of all collector diameters therefore lie on the same regression line as the drag coefficient of the various wires is approximately constant. At very high collector densities an additional quadratic term relative to the aerodynamic blockage effect is expected to appear, thus further decreasing the generated thrust. As for the thrust-to-power ratio, the maximum value obtained with collectors with a diameter of $0.03 \ \textrm{mm}$ is marginally lower than the case of Configuration I, due to a higher current consumption, driven by the different ignition voltage between the various configurations. Dimensions of the electrodes appear to have more influence on thrust density than on thrust-to-power ratio values. Within the tested configurations of the present study, the grid density is the primary parameter influencing the performance of the grid-based EAD thruster. However, due to its simplified definition, it does not fully capture higher-order phenomena, for which absolute values of diameters and spacings remain significant.

### Thrust-to-weight comparison

The radical change in the geometry of the collectors in the gridded thruster, compared to the configurations reported in the literature, requires an analysis in terms of thrust-to-weight ratio *T*/*W*—a crucial parameter in the aeronautical field, particularly for EAD thrusters (which are further disadvantaged by the additional weight of the high-voltage power converter required to achieve high operating voltages). Table 3 presents a comparison of the thrust-to-weight ratios for the optimal configurations identified in this study, alongside recent results from an optimization study involving streamlined airfoil collectors^21^. For the latter, two manufacturing approaches are considered: collectors made from a $0.1 \ \textrm{mm}$ thick aluminum sheet shaped as an airfoil—referred to as *shell*—and a prototyped configuration consisting of 3D-printed airfoils made of ASA with a $25\%$ infill, coated with a $0.025 \ \textrm{mm}$ aluminum foil. In all cases, only the mass of the collectors is included in the analysis. The weight contribution of the emitters is considered negligible due to their relatively low density, and the mass of the structural supports and frame is also neglected, as the objective is to enable comparisons independent of the test rig, which was designed solely for experimental purposes and does not represent the final version of the thruster. It is worth noting that, although conventional profile-based collectors offer superior structural properties compared to wires, the compactness afforded by the latter—owing to their negligible size along the thrust direction—allows weight savings through light support structures, making a meaningful comparison with the state of the art possible. The table reveals substantial differences between grid structures and airfoil collectors, with thrust-to-weight ratios reaching a maximum of 9.348 for Configuration I and 9.513 for the $d_e=0.03$, $d_c=0.03 \ \textrm{mm}$ configuration. These values correspond to improvements of $1877\%$ and $1912\%$, respectively, over the prototyped airfoil collector. These findings demonstrate the substantial advantages of grid-based collector geometries in terms of thrust-to-weight performance, enabling the design of highly compact propulsive units with minimal volume.

**Table 3:** Thrust-to-weight comparison between grid-based collectors and state of the art thruster.

		$d_e$ [mm]	$d_c$ [mm]	$\rho _e$ [-]	$\rho _c$ [-]	$\mathrm {T/W}$
Grids		0.03	0.10	0.002	0.080	9.348 (+1877%)
		0.10	0.10	0.006	0.053	6.228 (+1217%)
		0.03	0.03	0.002	0.032	9.513 (+1912%)
		0.03	0.20	0.002	0.080	8.295 (+1655%)
Ref.^21^	Shell					0.696 (+47%)
	Prototyped					0.473

## Conclusions

The present study examined the use of grid-based collectors to enhance the performance of electroaerodynamic (EAD) thrusters, aiming to improve thrust efficiency and density compared to the established airfoil-shaped collectors. Investigation of how wires diameter and spacings influence the performance revealed a significant increase in the thrust density coefficient, leading to an improvement by a factor of 2.14 (for Configuration I) for this new layout with respect to the state of the art^21^, without compromising the thrust-to-power ratio, which is comparable to that of traditional airfoil collectors. Collector mesh density was identified as the primary parameter influencing performance. An optimal range between 0.04 and 0.08 was determined, beyond which further increases lead to diminishing performance due to elevated aerodynamic drag. The emitter mesh density was found to play a secondary role: a density of 0.002 produced the best results for high-density collector configurations, as a result of a trade-off between the amount of generated ions and electrostatic shielding. Variations in emitter and collector diameters were found to affect performance, allowing the identification of the optima for each performance parameter. Smaller-diameter emitters generated higher thrust densities, whereas larger-diameter collectors enabled greater mesh densities. Reverse corona effects were associated with increased current consumption and reduced net thrust, particularly at lower collector densities. The thrust-to-weight ratio obtained by the grid configurations revealed promising improvements when compared to the traditional implementations (implying also a significant reduction in system volume), further supporting the adoption of this layout on flying platforms. Dimensional results show a maximum thrust density of $7.03 \ \mathrm {N/m^2}$ for a single stage unit, achieved with the best geometry of Configuration III (emitters spacing: $15 \ \textrm{mm}$, collectors spacing: $2.5\ \textrm{mm}$) at $20 \ \textrm{kV}$ of applied voltage, roughly double that of the thruster used in the MIT airplane^7^ and the ionocraft^9^, accounting for the different characteristics (i.e., voltage and gap). Note that the former is a twin stage design, therefore the performance increase for a single stage is expected to be higher. The highest thrust-to-power value has been obtained for Configuration I with emitters spacing $15 \ \textrm{mm}$ and collectors spacing $2.5 \ \textrm{mm}$, reaching a value of $6.52 \ \mathrm {N/kW}$, slightly higher than that found in the above-mentioned works^7,9^, which stood at $6.2 \ \mathrm {N/kW}$ and $6.4 \ \mathrm {N/kW}$, respectively. The findings suggest that grid-based EAD thrusters offer substantial promise for the development of efficient, lightweight and sustainable propulsion systems, with the potential to drive significant advancements in electric propulsion technologies.

## Data Availability

All data required to reproduce the results and figures presented in this paper are publicly available at Zenodo under the DOI:  10.5281/zenodo.17395632.
