# Correction: Atmospheric electroaerodynamic thrusters with grid collectors

**DOI:** 10.1038/s41598-026-47570-y

**Published:** 2026-04-03

**Authors:** Raffaello Terenzi, Stefano Trovato, Davide Usuelli, Marco Belan

**Affiliations:** Department of Aerospace Science and Technology, Politecnico di Milano, 20156 Milan, Italy

Correction to: *Scientific Reports* 10.1038/s41598-025-25308-6, Published online 24 November 2025.

The original version of this Article contained errors in the names of authors Raffaello Terenzi, Stefano Trovato, Davide Usuelli & Marco Belan which were incorrectly given as Terenzi Raffaello, Trovato Stefano, Usuelli Davide & Belan Marco.

The original Article has been corrected.

